# Hyaluronic acid impacts hematological endpoints and spleen histological features in African catfish (*Clarias gariepinus*)

**DOI:** 10.1186/s12917-024-04113-9

**Published:** 2024-07-05

**Authors:** Mohamed Hamed, Mohamed H. Kotob, Nasser S. Abou khalil, Esraa A. Anwari, Walaa Bayoumie El Gazzar, Shaimaa K. A. Idriss, Michel E. Fakhry, Amina A. Farag, Mahmoud S. Sabra, Sally M. Salaah, Souzan Abdel-Zaher, Fatma Alzahraa Yehia Saad, Mervat Naguib, Jae-Seong Lee, Alaa El-Din H. Sayed

**Affiliations:** 1 Department of Zoology, Faculty of Science, Al-Azhar University (Assiut Branch), Assiut, 71524 Egypt; 2 Department of Comparative Biomedical Sciences, School of Veterinary Medicine, Louisiana State University, Skip Bertman Drive, Baton Rouge, LA 70803 USA; 3 Department of Pathology, Faculty of Veterinary Medicine, Assiut University, Assiut, 71526 Egypt; 4 Division of Pharmacology and Toxicology, Department of Pharmaceutical Sciences, University of Vienna, Vienna, 1090 Austria; 5 Department of Animal Physiology and Biochemistry, Faculty of Veterinary Medicine, Badr University, Assuit, Egypt; 6 Department of Medical Physiology, Faculty of Medicine, Assuit University, Assiut, 71516 Egypt; 7 Department of Zoology, Faculty of Science, Assiut University, Assiut, 71516 Egypt; 8 Department of Anatomy, Physiology and Biochemistry, Faculty of Medicine, the Hashemite University, Zarqa, 13133 Jordan; 9 9Department of Medical Biochemistry and Molecular Biology, Faculty of Medicine, Benha University, Benha City, 13518 Egypt; 10 Department of Aquatic Animal Medicine and Management, Faculty of Veterinary Medicine, Assiut University, Assiut, 71516 Egypt; 11 Department of Medical Biochemistry and molecular biology, Faculty of Medicine, Assiut University, Assiut, 71516 Egypt; 12 Department of Forensic Medicine and Clinical Toxicology, Faculty of Medicine, Benha University, Benha City, 13518 Egypt; 13 Department of Pharmacology, Faculty of Veterinary Medicine, Assiut University, Assiut, 71516 Egypt; 14 Fresh Water Division, National Institute of Oceanography and Fisheries, NIOF, Cairo, Egypt; 15 Department of Molecular Biology, Molecular Biology Research & Studies Institute, Assiut University, Assiut, 71516 Egypt; 16 Department of Biotechnology, Molecular Biology Research & Studies Institute, Assiut University, Assiut, 71516 Egypt; 17 Department of Biological Sciences, College of Science, Sungkyunkwan University, Suwon, 16419 South Korea

**Keywords:** Hyaluronic acid, Fish, Erythrocytes, Micronuclei, Apoptosis, Spleen

## Abstract

Since its identification in the vitreous humour of the eye and laboratory biosynthesis, hyaluronic acid (HA) has been a vital component in several pharmaceutical, nutritional, medicinal, and cosmetic uses. However, little is known about its potential toxicological impacts on aquatic inhabitants. Herein, we investigated the hematological response of *Clarias gariepinus* to nominal doses of HA. To achieve this objective, 72 adult fish were randomly and evenly distributed into four groups: control, low-dose (0.5 mg/l HA), medium-dose (10 mg/l HA), and high-dose (100 mg/l HA) groups for two weeks each during both the exposure and recovery periods. The findings confirmed presence of anemia, neutrophilia, leucopoenia, lymphopenia, and eosinophilia at the end of exposure to HA. In addition, poikilocytosis and a variety of cytomorphological disturbances were observed. Dose-dependent histological alterations in spleen morphology were observed in the exposed groups. After HA removal from the aquarium for 2 weeks, the groups exposed to the two highest doses still exhibited a notable decline in red blood cell count, hemoglobin concentration, mean corpuscular hemoglobin concentration, and an increase in mean corpuscular volume. Additionally, there was a significant rise in neutrophils, eosinophils, cell alterations, and nuclear abnormalities percentages, along with a decrease in monocytes, coupled with a dose-dependent decrease in lymphocytes. Furthermore, only the highest dose of HA in the recovered groups continued to cause a significant increase in white blood cells. White blood cells remained lower, and the proportion of apoptotic RBCs remained higher in the high-dose group. The persistence of most of the haematological and histological disorders even after recovery period indicates a failure of physiological compensatory mechanisms to overcome the HA-associated problems or insufficient duration of recovery. Thus, these findings encourage the inclusion of this new hazardous agent in the biomonitoring program and provide a specific pattern of hematological profile in HA-challenged fish. Further experiments are highly warranted to explore other toxicological hazards of HA using dose/time window protocols.

## Introduction

Industrial growth and the expanding population around rivers are posing an alarming danger to freshwater ecosystems [1]. Because new chemicals are constantly being introduced into the aquatic ecosystem, research on the consequences of pollution and its effects on fish biological aspects is indispensable [2]. Human dietary intake of fish muscle carrying synthetic contaminants, which have been responsible for a number of health problems, makes this issue crucial for human health as well [1].

Hyaluronic acid (HA) is a water soluble high molecular weight molecule (up to 10MDa), it is the main constituent of the extracellular matrix. Composed of a longitudinal unbranched mucopolysaccharide chain, alternating glucuronic disaccharide and N-acetylglucosamine units. HA belong to the glycosaminoglycan family (GAGs); However, HA is generally not covalently associated with protein and the only non-sulfate GAG. The HA molecule may occur in several forms, size, and architectures, under ideal physiological conditions HA is characterized by a random coil structure. It can either move freely or be present in a tissue state. owing to its adaptable biological nature and significant viscoelastic qualities [3].

It is commonly used in topical cosmetic products, biomedical applications, and tissue remodeling [4–6]. Numerous cited articles have confirmed the safety and tolerability of HA in various animal species [7]. However, a close examination of the literature reveals a lack of academic research focusing on its toxicity in aquatic creatures. The physicochemical characteristics of HA-based preparations depend entirely on its molecular weight [8]. Hyaluronic acid (HA) with high molecular weight (HMW) demonstrates anti-inflammatory and immunosuppressive properties in synoviocytes of a rabbit model of osteoarthritis, whereas low molecular weight (LMW) HA functions as a potent pro-inflammatory agent in murine alveolar macrophages [8]. Sodium hyaluronate’s strong water-binding ability and high density altered RBCs to ellipsoidal by osmotic dehydration during cryopreservation of sheep RBCs in vitro [9]. HMW HA increases suicidal tendencies in splenic T cells of C57BL/6 mice independently of Fas and caspase-mediated apoptotic pathways [10]. On the other side, an increased rigidity index and decreased aggregation tendency were observed in erythrocytes of rheumatoid arthritis patients incubated with HMW HA due to stabilization of the membrane cytoskeleton [10, 11]. HMW HA stimulated phagocytosis in polymorphonuclear neutrophils in vitro by interacting with CD44 receptors [12].

A study shown that the in vitro toxicity in zebra fish of lipid nanoparticles containing hexadecyltrimethylammonium bromide was reduced when they were coated with HA after their formation [13].

Furthermore, the zebrafish model demonstrates minimal toxicity of HA protamine nanocapsules [14]. Treating mice with LMW HA prevents neutrophil apoptosis through the PI3K/Akt1 pathway by increasing Mcl-1 expression, resulting in the initiation of lung inflammation [15]. LMW HA enhances macrophage activation by inducing the production of pro-inflammatory cytokines and chemokines [16].

Estimating blood parameter is a commonly employed sensitive technique for evaluating the toxicological impacts of pollution on fish [17–22]. Alterations in hematological outcomes offer a dependable means of assessing both fish health and their environment inexpensively, as even minor fluctuations in water physicochemical parameters can influence blood parameters [23–25].

The close relationship between fish and aquatic habitats provides an excellent opportunity for hematological indices to convey early responses to environmental challenges before negative effects become obvious [26]. Additionally, the detection of genotoxic and cytotoxic effects in the aquatic ecosystem is aided by tests for micronuclei (MN), nuclear abnormalities, and morphological erythrocyte changes (MEA) [2, 27]. When exposed to chemotoxicants, particularly, the proportion of micronuclei is a useful predictor of damage to the mitotic spindle machinery and chromosomal destruction [28, 29].

Among tropical farmed fish, the African catfish (*C. gariepinus*) is considered a notable species due to its rapid growth, ability to tolerate high stocking densities, consumer appeal, and resilience in environments with low oxygen levels and poor water quality [30–32]. In the context of environmental toxicology and bioremediation procedures, its consumption pattern and habitat on the bottom of water bodies make it an indispensable fish model [33, 34].

Thus, our experiments were performed to investigate the potential hematotoxic effects of serial concentrations of HA during a two-week exposure period and the same time window of recovery on *C. gariepinus*, employing hematological outcomes and morphological erythrocytic modifications as bio-indicators.

## Materials and methods

### Chemicals

Hyaluronic acid powder was purchased from Selleck Chemicals Company (Houston, TX, USA) with purity 99.63% (Catalog No. S5865). Stock solution of HA was prepared using distilled water according to the manufacturer’s instructions and stored at -80 °C.

### Fish

African catfish (*C. gariepinus*), weighing 200 ± 25 g and measuring 25 ± 5 cm, were obtained from the Aquaponic Unit of Assuit University, Assuit, Egypt. The fish were confirmed to be free from infections and diseases and transported to the Fish Biology and Environmental Pollution Lab at the Zoology Department, Faculty of Science, Assuit University, Egypt. The experiments adhered to the guidelines outlined in Test Guideline No. 203 for Fish Acute Toxicity Testing [35].

Prior to the experiments, the fish were acclimatized for 21 days in 120-L vessels filled with dechlorinated tap water. The water conditions were maintained at a temperature of 26 ± 2 °C, pH levels ranging between 7.2 and 7.6, oxygen saturation exceeding 80%, ammonia, nitrite, and nitrate levels were 0.06 ± 0.02, 0.01 ± 0.009, and 0.35 ± 0.13 mg/L, with a standard light/dark photoperiod. Commercial food containing 30% protein, fish meal, calcium, sodium chloride, vitamins, soybean meal, wheat bran, maize, crude protein, lipids, and crude fiber was given to the fish twice a day.

### Experimental design

The acclimatized fish were allocated into four categories, each consisting of 24 fish in triplicate. The first group served as the control without any treatment. The second group received a dose of 0.5 mg/L HA. The third group received a dose of 1 mg/L HA. The fourth group received a dose of 100 mg/L HA. The exposure period extended for 14 days, followed by a 14-day recovery period. The concentrations of HA were chosen based on observed toxic values determined by previous studies [36–38].

The experimental aquariums were filled with dechlorinated water, and the compounds were added before introducing the fish. This was done to reduce the potential decrease in the nominal dosing concentrations resulting from the adhesion of particulates or compounds to the glass. Following this, 50% of the tank’s water was replaced daily and promptly supplemented with HA to maintain the experimental doses. Six fish from each treatment group were individually selected and anesthetized using ice [39] to minimize stress after the two-week exposure phase and the subsequent 14-day recovery period. Subsequently, these fish underwent additional biochemical and histological testing [28].

### Hematological parameters

Following the HA exposure and recovery periods, six fish were randomly sampled from each trail. To minimize potential handling stress, the fish were anesthetized using ice [40]. Blood samples were obtained in sterile tubes by the perforation of caudal vein, part of blood specimens collected in heparinized tubes for hematological investigation, while the other part was allowed to coagulate for twenty minutes at room temperature, then centrifuged at 5000 rpm. The obtained blood serum and kept at 4 °C for further biochemical investigations.

Heparinized blood sample was used to quantify ed blood cells (RBCs, 10^6/mm³) using Dacie’s diluting fluid in conjunction with a Thoma hemocytometer chamber. The hematocrit ratio (Hct, %) was determined using a capillary hematocrit tube, and the hemoglobin concentration (Hb, g/dL) was quantified using the cyanomethemoglobin technique [41] by spectrophotometry at 540 nm. Mean corpuscular volume (MCV, µm³), mean corpuscular hemoglobin (MCH, pg), and mean corpuscular hemoglobin concentration (MCHC, %) were calculated based on established equations.

MCV (µm^3^) = [(Hct, %) ×10] / (RBC× Million/ µL).

MCH (pg) = [(Hb, g/dL) ×10] / (RBC× Million/ µL).

MCHC (%) = [(Hb, g/dL) ×100] / (Hct, %).

Under oil immersion and at ×100 magnification, differential leukocytes in May-Grunwald-Giemsa-stained peripheral blood smears were investigated. White blood cells (WBCs, 10^3/mm³) were counted using the protocol described by Knight et al. (2011). From 100 leukocytes per slide, lymphocytes, neutrophils, and monocytes were identified [42].

### Micronucleus test and erythrocytes alterations

To detect micro-nucleated and morphologically changed erythrocytes, part of the whole blood sample was preserved in absolute methanol, stained with hematoxylin and eosin, and evaluated under a microscope according to predetermined features [43, 44].

### Erythrocyte programmed cell death (apoptosis)

Using the technique outlined by Sayed [45], apoptosis was identified using acridine orange staining, and red blood cells were examined under a fluorescence microscope (Zeiss Axioplan2) equipped with a digital 3 CCD color video camera (Sony, AVTHorn, Japan).

### Histological processing

Specimens from the spleens of catfish from all groups were obtained and preserved in 10% neutral-buffered formalin. Following fixation, the samples underwent dehydration through escalating ethanol concentrations and were subsequently cleared in xylene. The specimens were then embedded in paraffin wax. Sections with a thickness of 5 μm were sliced from the paraffin-embedded samples and stained using the following histological stains:1 -Hematoxylin and Eosin stain for a comprehensive histological examination [46].

2-Masson trichrome stain for connective tissue staining according to [47]. The stained sections were later examined using a light microscope (Olympus, USA) by a histologist who was blinded to the groups.

### Statistical analysis

The findings were presented as mean ± standard error (SE). One-way analysis of variance (ANOVA) and Duncan post-hoc test was used to analyze the data using SPSS software version 16 (SPSS Inc., Chicago, USA). A difference was considered statistically significant when *p* < 0.05.

## Results

### The effects of different concentrations of hyaluronic acid on the hematological parameters

**Fig. 1:**
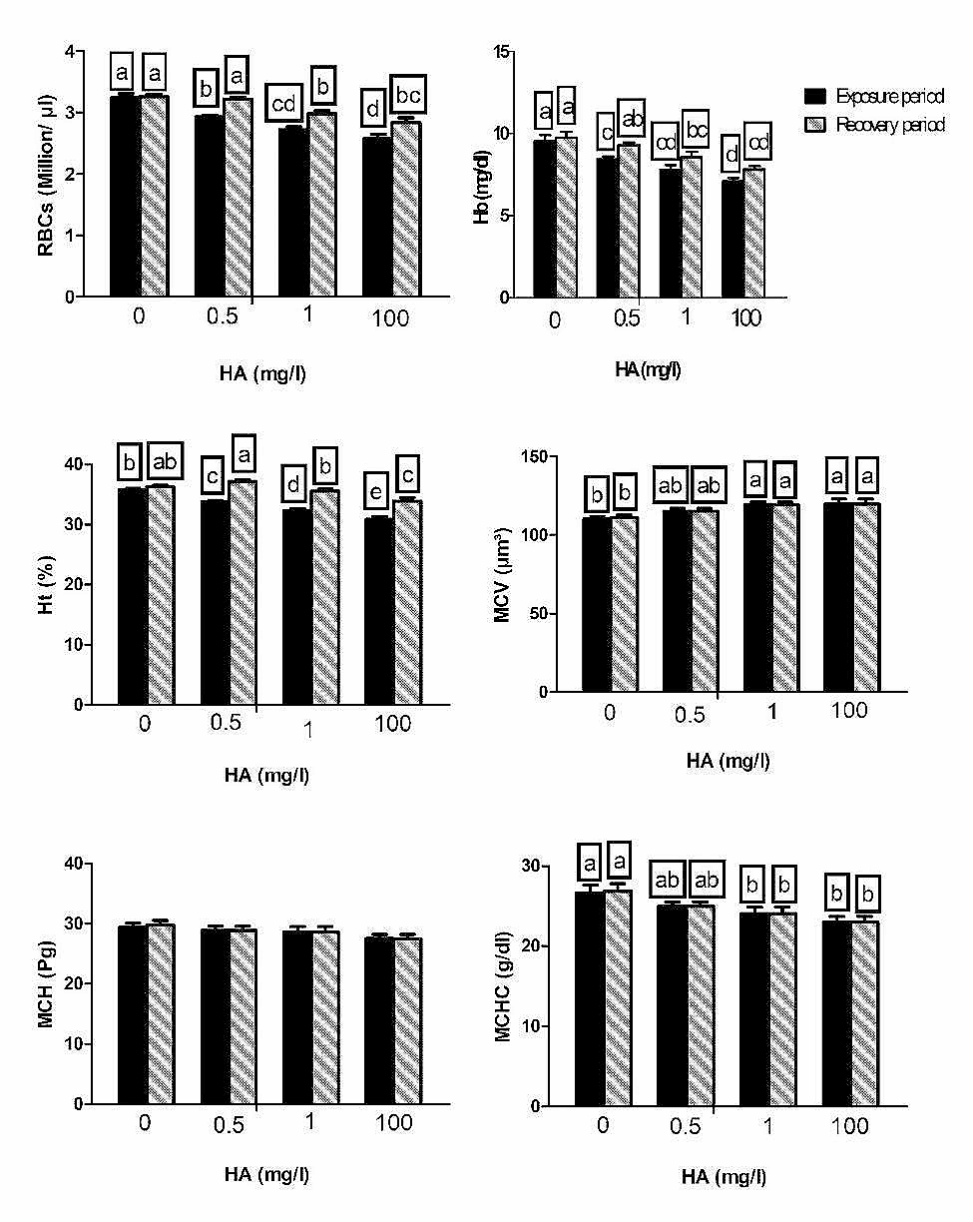
The erythrocyte-associated indicators in *C. gariepinus* exposed to the effects of different doses of HA (0.5, 1, and 100 mg/L) for 14 days and recovered for 14 days. RBCs: red blood cells; Hb: hemoglobin; Ht: hematocrit; MCV: mean corpuscular volume; MCH: mean corpuscular hemoglobin; MCHC: mean corpuscular hemoglobin concentration. Bars represent means ± SE of 6 fish/group. Different letters indicate significant differences among treatments (*p* < 0.05)

Figure 1 shows the effects of nominal concentrations of HA during exposure and recovery periods on the erythrocyte-associated indicators. RBC count and Hb content significantly decreased in HA-exposed groups. HA at a concentration of 0.5 mg/L reduced these outcomes to a lesser extent than HA at the other concentrations. Following withdrawal of HA from the aquarium for 2 weeks, the groups previously exposed to the highest two doses still exhibited a significant reduction in the number of RBCs and Hb concentration, while the low-dose group returned to control levels. A dose-related drop in Ht percent was observed in the challenged groups. Only the highest dose of HA in the recovered groups still caused a significant increase in Ht percent compared to the control. HA at doses of 1 and 100 mg/L in both exposed and recovered groups increased MCV and reduced MCHC to the same degree of potency, without significant change following exposure to 0.5 mg/L. However, insignificant alterations were found in MCH in all experimental groups compared to the control.

**Fig. 2:**
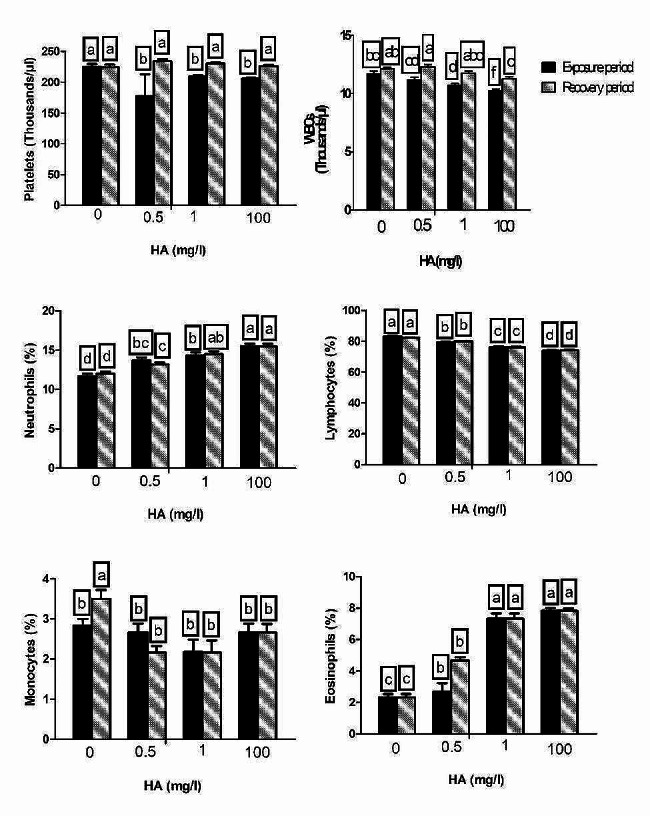
The haematological cellular components other than RBCs in *C. gariepinus* exposed to the effects of different doses of HA (0.5, 1, and 100 mg/L) for 14 days and recovered for 14 days. WBCs: white blood cells. Bars represent means ± SE of 6 fish/group. Different letters indicate significant differences among treatments (*p* < 0.05)

Figure 2 shows the effects of nominal concentrations of HA during exposure and recovery periods on haematological cellular components other than RBCs. Neutrophilia, leucopenia, lymphopenia, and eosinophilia are distinguishing cytopathological features in the experimental groups at all doses after the intervention periods. White blood cell (WBC) counts in fish supplemented with the high dose were significantly lower than those supplemented with the low dose. The reduction in WBC count in the medium-dose group was insignificantly different from that in the low-dose group. WBCs in the fish translocated to HA-free water were normalized, except in those pre-challenged with the high dose. During the intervention phase, neutrophils were significantly greater in the high-dose group than in the other intoxicated groups. The percentage of neutrophils in the low-dose group did not exhibit a significant difference from the medium-dose group. Even after culturing the fish in clean aquaria for two weeks following the exposure period, the percentage of neutrophils in all recovered groups still showed a significant increase. The medium- and high-dose groups exhibited an equipotent increase in the neutrophil count, which was significantly higher than that observed in the low-dose group. A dose-related decrease in lymphocytes was observed in both intervention and recovery stages. The monocyte percentage insignificantly changed during the exposure period. Surprisingly, HA-associated monocytopenia was observed following the culture of fish for two weeks in clean aquaria after the intervention. The medium and high doses of HA were responsible for an equipotent increase in eosinophils, and the response pattern caused by these doses was significantly greater than that caused by the small dose. The exact modulatory style was present in the recuperation stage. Platelet count showed an equipotent decrease in the exposed groups, and translocation of fish to HA-free water succeeded in normalizing this cytohematological abnormality.

### Effects of different concentrations of hyaluronic acid on the erythron profiles

**Fig. 3:**
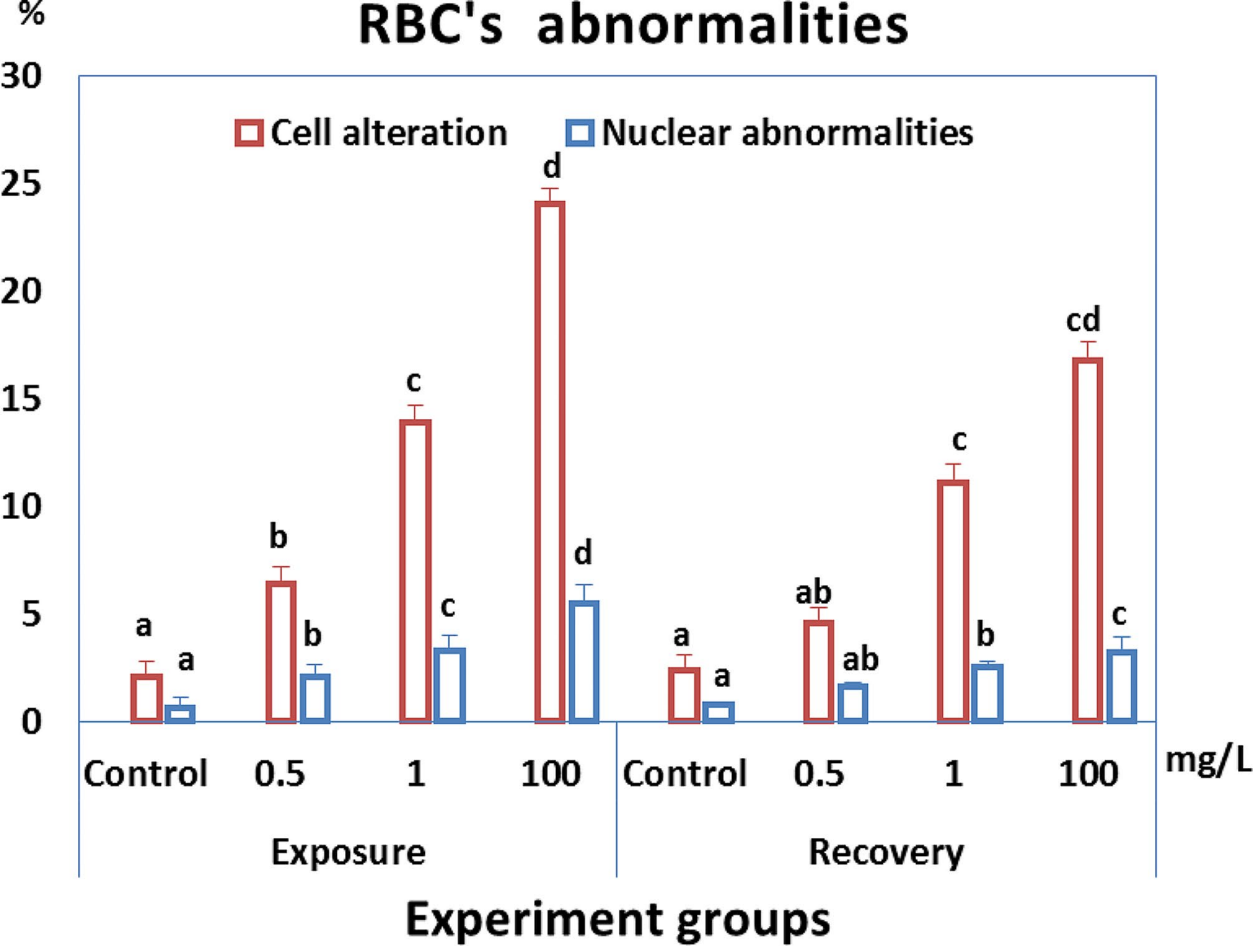
The percentage of cell alteration and nuclear abnormalities of RBCs in *C. gariepinus* exposed to the effects of different doses of HA (0.5, 1, and 100 mg/L) for 14 days and recovered for 14 days. Bars represent means ± SE of 6 fish/group. Different letters indicate significant differences among treatments (*p* < 0.05)

**Fig. 4:**
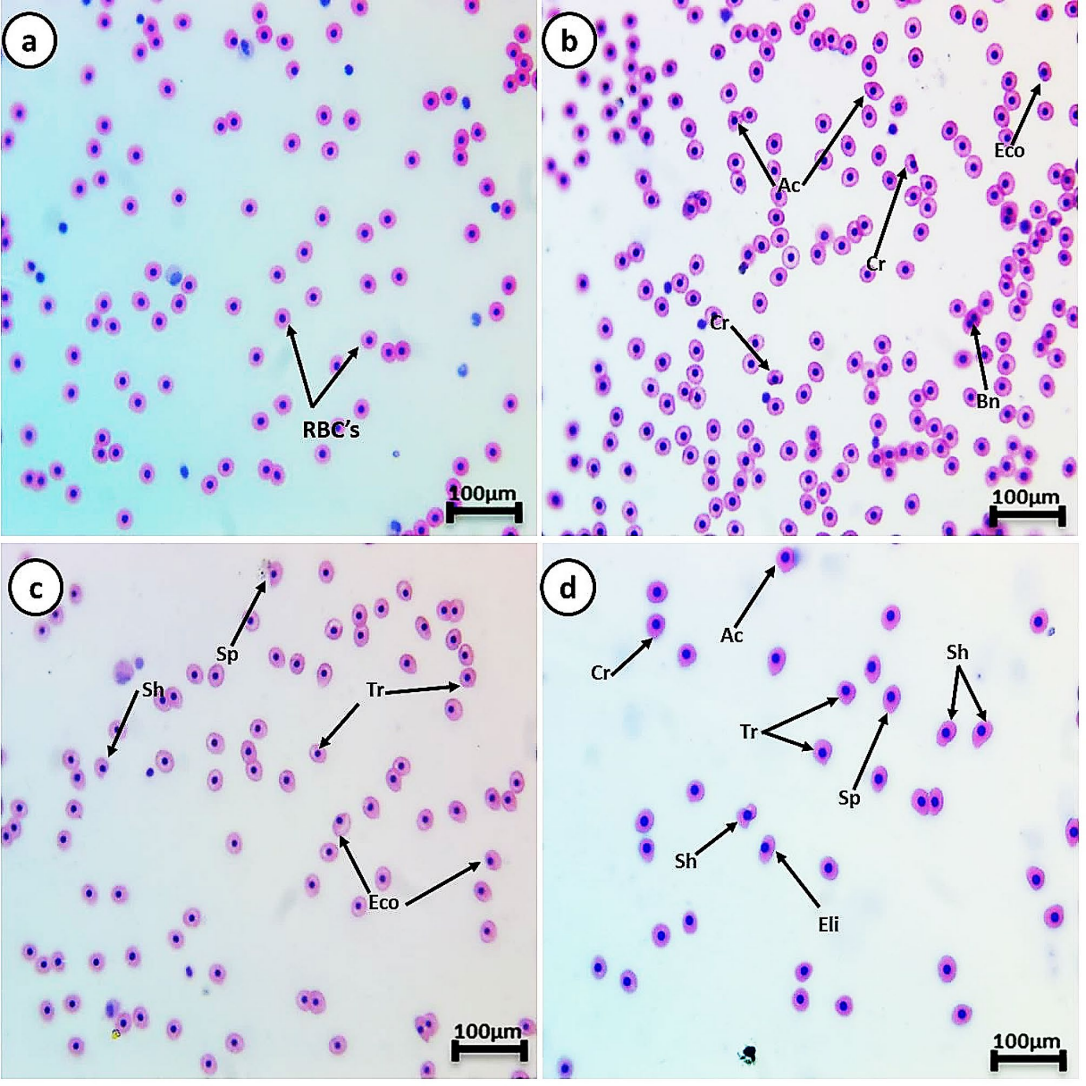
Represented blood smears from of *C. gariepinus* showing (**a**) the normal erythrocytes, (**b**) the deformed ones after exposure to 0.5 mg/L HA, (**c**) the deformed ones after exposure to 1 mg/L HA, and (**d**) 100 mg/L HA; Tr, tear-drop cell; Sp, spinocyte; Cr, crenated cell; Ac, acanthocyte; Eco, eccentric nucleus; Bn, Bionucleus; Kn, kidney shape; Sh, schistocytic and Eli, Eliboat shape (H & E stain, scale bar: 100 μm)

**Fig. 5:**
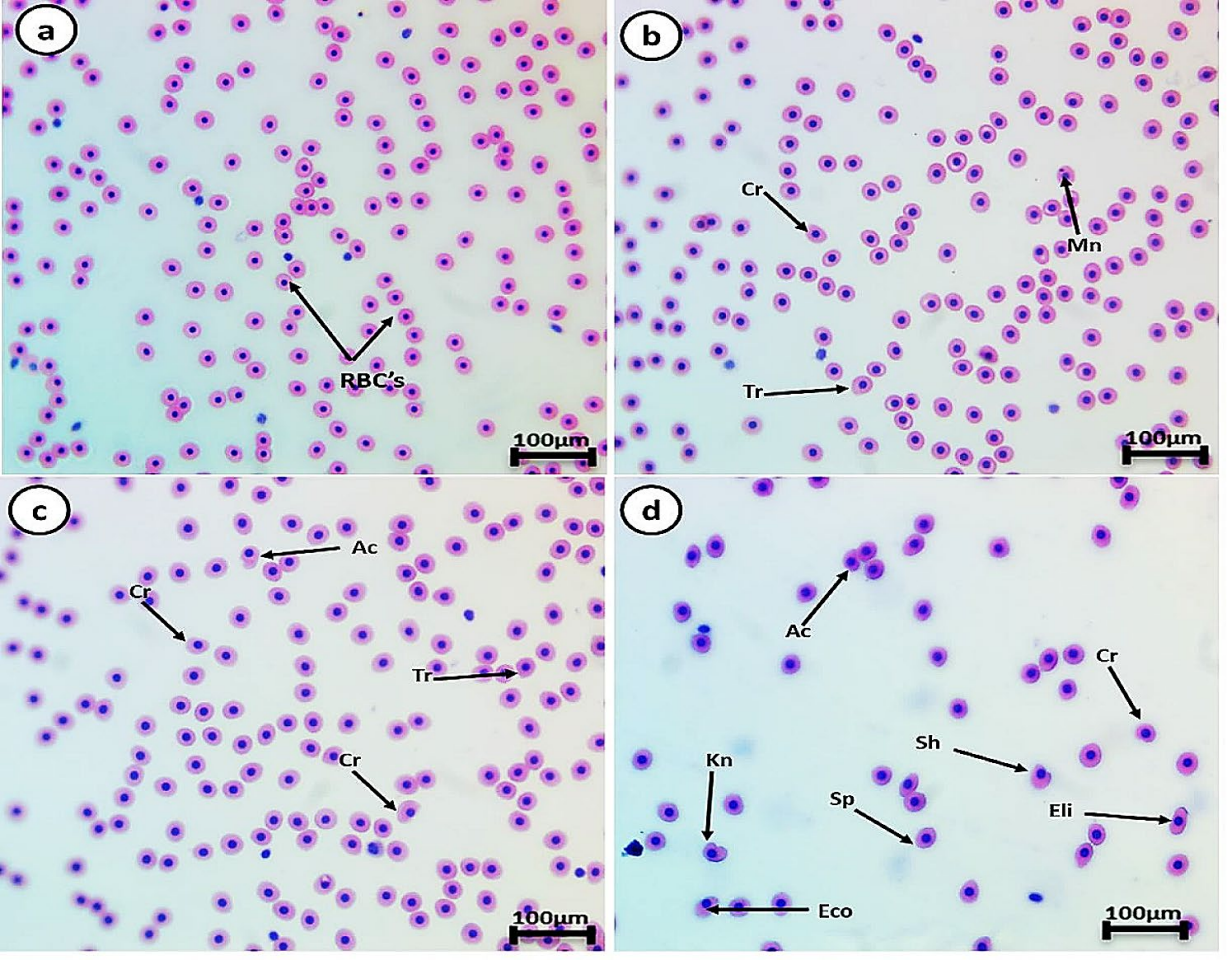
Represented blood smears from of *C. gariepinus* showing (**a**) the normal erythrocytes, the deformed ones after post-exposures (Recovery) (**b**) 0.5 mg/L, (**c**) 1 mg/L, and (**d**) 100 mg/L HA; Tr, tear-drop cell; Sp, spinocyte; Cr, crenated cell; Ac, acanthocyte; Eco, eccentric nucleus; Mn, micronucleus; Kn, kidney shape; Sh, schistocytic and Eli, Eliboat shape (H & E stain, scale bar: 100 μm)

The control group’s RBCs had a typical form with a nucleus in the center. Smears from all HA-exposed groups displayed poikilocytosis in RBCs after both exposure and recovery periods. The cells showed varied morphological alterations, including tear-drop cells, spinocytes, crenated cells, acanthocytes, cells with eccentric nucleus, bi-nucleated cells, kidney-shaped cells, schistocytes, and elliptocyte shapes (Figs. 3 and 4, and 5). The percentages of cell alterations and nuclear abnormalities in RBCs significantly increased in a dose-dependent manner in the exposed groups (Fig. 3). Compared to the control group, the percentages of observed alterations after the 14-day exposure period were 6.4%, 13.9%, and 24.1% for cell alterations, and 2.1%, 3.3%, and 5.5% for nuclear abnormalities after exposure to 0.5, 1, and 100 mg/L HA, respectively. The percentage of cell alterations in the recovered groups previously exposed to 1 and 100 mg/L HA was significantly greater than in the control group, with no significant difference between them. The recovered groups previously exposed to 1 and 100 mg/L HA showed a significant increase in the percentage of nuclear abnormalities compared to the control. The percentage of nuclear abnormalities in the recovered group exposed to 100 mg/L HA was significantly greater than in the group exposed to 1 mg/L HA. Following the recovery period, the percentages of observed cell alterations were 4.6%, 11.1%, and 16.8% for cell alterations, and the percentages of nuclear abnormalities were 1.6%, 2.5%, and 3.2% after exposure to 0.5, 1, and 100 mg/L HA, respectively.

### Effects of different concentrations of hyaluronic acid on HA mediated eryptosis

**Fig. 6:**
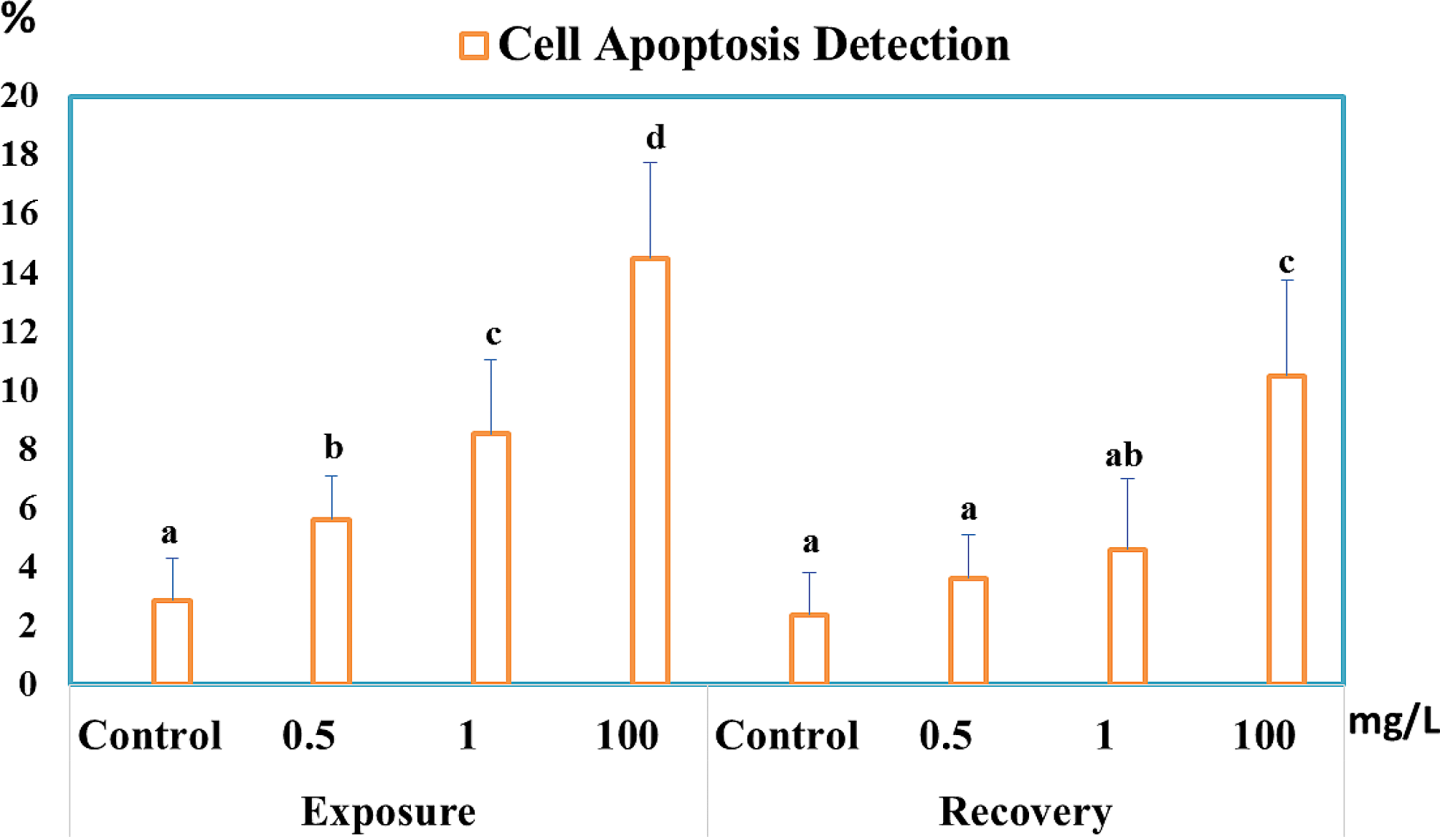
Percentage of apoptotic cells of *C. gariepinus* after 14 days of exposure to 0.5, 1, and 100 mg/L HA, and 14 days of recovery to the same doses. Data are presented as mean + SE of 6 fish/group. Bars with different superscript letters are significantly different at *P* < 0.05

In a concentration-dependent manner, the proportion of apoptotic cells rose noticeably in the HA-exposed groups compared to the control group. The percentages of observed apoptosis were 5.62%, 8.53%, and 14.5% after exposure to 0.5, 1, and 100 mg/L HA, respectively (Fig. 6). After the withdrawal phase, the proportion of apoptotic RBCs remained greater in the highest dose group than in the control group. The percentages of observed apoptosis during the recovery period were 3.62%, 4.6%, and 10.5% for the groups previously exposed to 0.5, 1, and 100 mg/L HA, respectively (Fig. 6).

### Histological findings

**Fig. 7:**
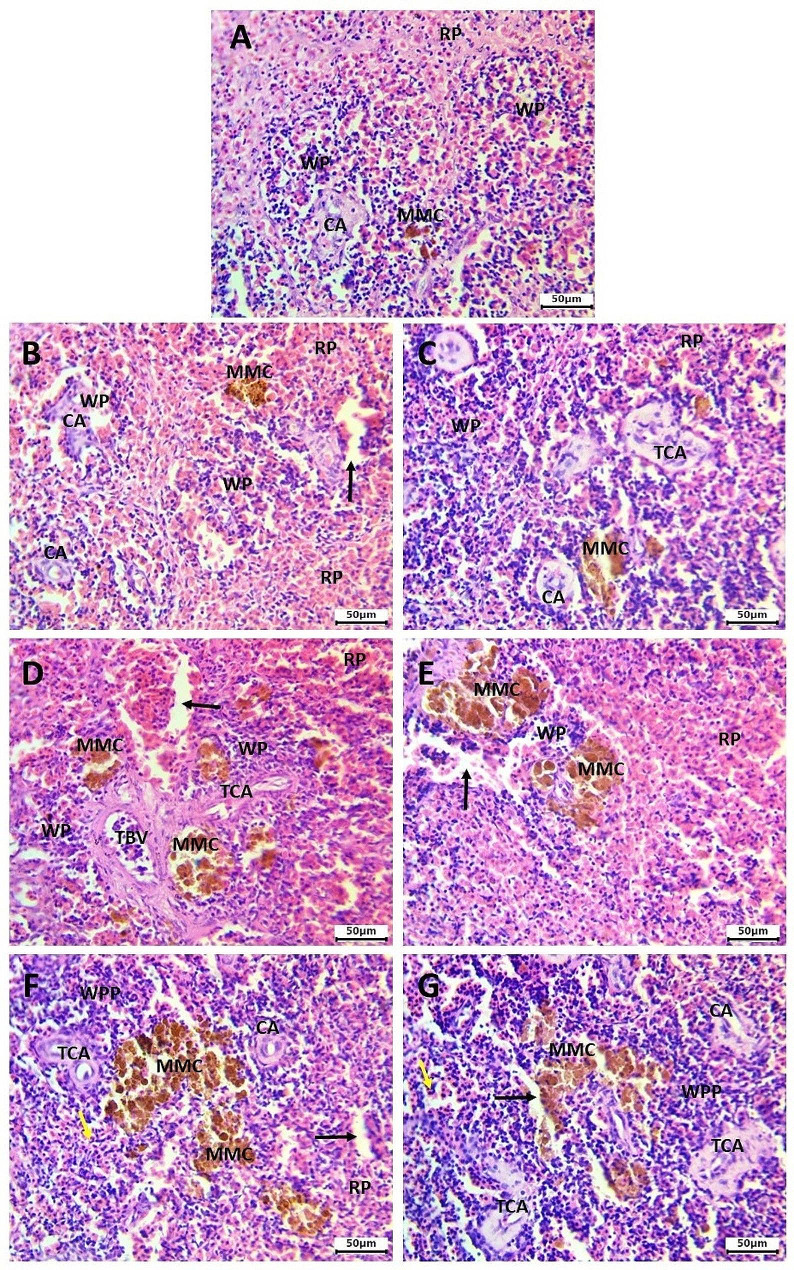
The spleen of *C. gariepinus* stained with H&E stain showing the normal histological structure of the control group (**A**). Mild histological alterations in spleen of 0.5 mg/L HA exposed (**B**) and recovered (**C**) groups. Moderate changes with the increase in size and amount of melanomacrophage centers in spleen of 1 mg/L HA exposed (**D**) and recovered (**E**) groups. Pronounced histological changes with greater amount of melanomacrophae centers, thickened wall of blood vessels and trabeculae, increased sinusoidal space and white pulp proliferation in spleen of 100 mg/L HA exposed (**F**) and recovered (**G**) groups. Black arrow refers to increased sinusoidal spaces, neutrophilic infiltration (yellow arrow), white pulp (WP), white pulp proliferation (WPP), red pulp (RP), melanomacrophag center (MMC), central arteriole (CA), thickened clogged central arteriole (TCA), thickened clogged blood vessel (TBV). Bar = 50 μm

In fish from the control group, the spleen exhibited a normal histological structure, characterized by white pulp with aggregated lymphoid cells, red pulp consisting of an interconnected system of splenic cords, and sinusoid capillaries filled with red blood corpuscles. Additionally, there were lymphatic cells and ellipsoid structures indicative of terminal capillaries. These structures featured a thin endothelial cell layer composed of cubic cells, characterized by centrally positioned rounded nuclei. They were enveloped by a sheath of acidophilic fibrous connective tissues and accompanied by a series of aggregated lymphocytes. Moreover, the spleen exhibited several sizable melanomacrophage centers housing macrophage cells, in addition to numerous scattered smaller centers containing pigments spanning from yellow to dark brown (Fig. 7A).

The severity of the histological changes was dose-dependent in both the exposed and recovered groups following exposure to HA.

The spleen of catfish exposed to a low dose of HA (0.5 mg/L) and the recovered group showed mild histological alterations, with a slight increase in the number of melanomacrophage centers and increased sinusoidal space compared to that observed in the control spleen (Fig. 7B, C).

The spleen of *C. gariepinus* exposed to a medium dose of HA (1 mg/L), as well as the recovered group, exhibited moderate histological alterations characterized by an increase in the number and size of melanomacrophage centers around the blood vessels. These alterations were accompanied by perivascular fibrosis, resulting in thickening and clogging of the blood vessel, widening the sinusoidal space and clogged arteries (Fig. 7D, E).

The spleen of *C. gariepinus* exposed to a high dose of HA (100 mg/L) exhibited severe histological alterations, including an increase in the number and size of melanomacrophage centers. Connective tissue proliferation was observed around the walls of blood vessels, infiltrating the parenchyma of both red and white pulp. Additionally, splenic trabeculae were thickened. Degenerative and necrotic changes, white pulp proliferation, widening sinusoidal space and clogged arteries were clearly noticed (Fig. 7F, G). Splenitis and neutrophil infiltration was also detected, replacing the depleted lymphocyte count in the white pulp area. A decrease in the percentage of RBCs in the red pulp was observed (Fig. 7F, G). These histological changes persisted in the recovered group previously exposed to 100 mg/L HA, and to a lesser extent, but still remained severe compared to the control and low-dose exposed groups.

**Fig. 8:**
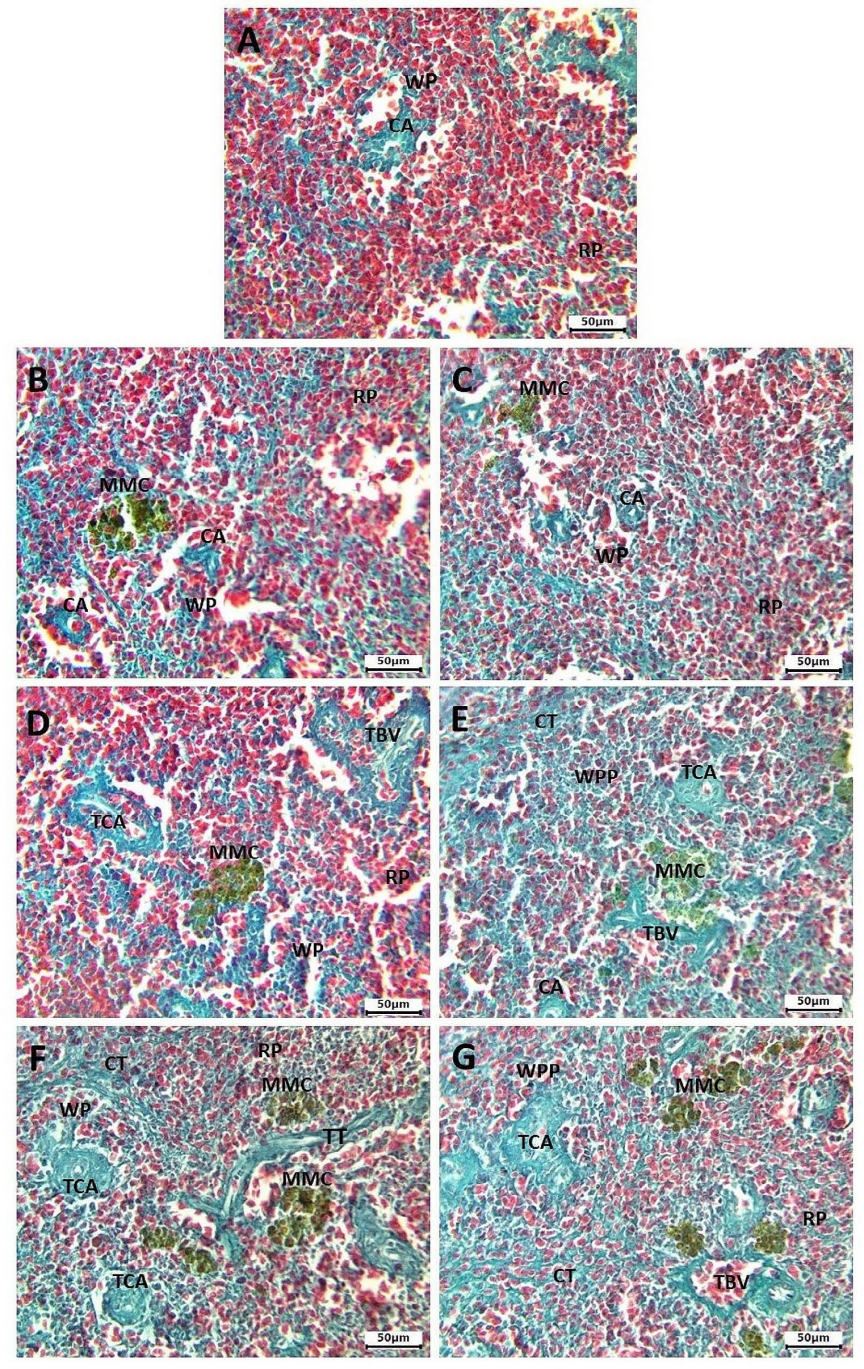
The spleen of *C. gariepinus* stained with masson trichrome stain showing the normal histological structure of the control group (**A**). Slight connective proliferation in spleen of 0.5 mg/L HA exposed (**B**) and recovered (**C**) groups. Moderate connective tissue proliferation with thickened wall of blood vessels in spleen of 1 mg/L HA exposed (**D**) and recovered (**E**) groups. Massive connective tissue proliferation with thickened wall of blood vessels and trabeculae in spleen of 100 mg/L HA exposed (**F**) and recovered (**G**) groups. White pulp (WP), white pulp proliferation (WPP) red pulp (RP), melanomacrophage center (MMC), thickened trabeculae (TT) connective tissue (CT), central arteriole (CA), thickened clogged central arteriole (TCA), thickened clogged blood vessel (TBV). Bar = 50 μm

The Masson trichrome stain revealed proliferation of connective tissue around the walls of blood vessels and splenic trabeculae, leading to their thickening. The extent of connective tissue proliferation was dose-dependent, with severity notably increased in the high-dose exposed and recovered groups compared to the control and other HA-exposed groups (Fig. 8A-G). Histological lesion scoring for different doses is presented in Table 1.

**Table 1:** Histological lesion scoring for different dose exposure to hyaluronic acid

	Control	Low-dose (0.5 mg/l HA)		Medium-dose (10 mg/l HA)		High-dose (100 mg/l HA)	
		Exposed	Recovered	Exposed	Recovered	Exposed	Recovered
Splenic necrosis	-	-	-	+	+	++	++
Melanomacrophage centers	-	+	+	++	++	+++	+++
Perivascular fibrosis	-	+	+	++	++	+++	+++
Thickened Trabeculae	-	-	-	++	++	+++	+++
Increased sinusoidal space	-	+	+	+	+	++	++
Neutrophilic infiltration	-	+	+	+	+	++	++
Clogged blood vessels	-	-	-	+	+	++	++
White pulp proliferation	-	-	-	+	+	++	++

(-) considered no change; (+) mild change; (++) moderate change; (+++) severe change

## Discussion

The HA-exposed groups exhibited an increased tendency toward anemia, depletion in lymphocytic and phagocytic cell populations, poikilocytosis, and various morphological alterations. The pattern of anemia was most remarkable in the highest dose group, and the percentages of cellular modifications and nuclear abnormalities in RBCs notably increased in a dosage-dependent manner. Although there was an enhancement in the investigated parameters following the recovery period, it was still to a lesser extent than in the corresponding intoxicated groups.

The observed reduction in RBC count could result from an increased haemolysis rate. The osmolality of the solution increases owing to strong water-binding affinity of HA, leading to intracellular dehydration and destruction of sheep RBCs in vitro [9]. HA also promotes calcium accumulation in the intracellular environment [37], strengthening the apoptotic signals and encouraging the cell suicidal decisions [48]. Although there is speculation that HA may degrade the pronephros, potentially leading to reduced erythrocyte production and anemia, no studies have yet confirmed this hypothesis. Conversely, culture of HA with RBCs of rheumatoid arthritis patients reduces membrane deformability and raised its rigidity index in a dosage-related way [10]. The drop in the number of RBCs and Hb content compromises the oxygen-delivering potential of the blood, causing tissues to suffer from hypoxia [28]. The decrease in RBC count and Hb concentration at the same proportion results in an insignificant change in MCH.

HA, a constituent of the extracellular matrix, regulates changes in cell volume during cardiovascular morphogenesis [49]. Additionally, HA-DNA superpolymers cause an increase in the size of HeLa cells [50]. The overproduction of reactive oxygen species under the burden of HA compromises the functional activity of the sodium-potassium pump in breast cancer cell lines [51]. Considering that this electrogenic pump plays an essential role in stabilizing cell volume against osmotic forces driving water influx, the loss of its activity may lead to fluid influx and could predispose to an increase in mean corpuscular volume observed following HA exposure and recovery.

Hyaluronic acid has the ability to regulate the activity of genes that are involved in the natural defense against viruses, as well as genes related to metabolism, during the early developmental stages of Atlantic salmon [52]. This outcome is compatible with the adverse effects of HA on the phagocytic potential of guinea-pig peritoneal macrophages in vitro due to its high viscosity, which interferes with cell membrane fluidity The percentage of neutrophils increased in both exposed and recovered groups. Considering that LMW HA stimulates the production of proinflammatory cytokines and chemokines [53], and neutrophils play a key role in initiating and regulating acute inflammatory responses in fish [54], it is suggested that HA promotes inflammatory reactions. HA deactivates monocytes mediated through engagement with CD44 and toll-like receptor 4 [33].

In the current model, HA-exposed groups were characterized by leukopenia, which persisted in the highest dose group following recovery. This observation can be explained by the fact that HA can induce apoptosis in splenic T lymphocytes of C57BL/6 mice through processes that are independent of both Fas and caspase [10]. The reduction in WBC count renders the body more vulnerable to infection.

Eosinophilia mediated by HA was evident in both exposed and recovered groups. HA increases the expression of hemopoietic growth factors by human peripheral blood eosinophils in vitro, resulting in in an extended lifespan [55]. Moreover, it promotes the growth and differentiation of CD34 + precursor cells purified from undifferentiated umbilical cord blood cells into fully developed eosinophils [56].

The impact of sodium HA on pleural adhesions and fibrosis in a rabbit model of experimental empyema was assessed in a previous report [38]. The authors observed a significant decrease in leukocyte count compared to the control group, which consistent with our findings.

A dose-dependent increase in the number of apoptotic RBCs was observed in this study, persisting even after the recovery period in the highest dose group. This finding corresponds to results found in a bladder cancer cell line incubated with sulfated HA fragments [57]. The latter induced cytotoxicity by stimulating pro-apoptotic mediators, increasing the transcript abundance of death receptor signaling proteins, and decreasing the gene expression of anti-apoptotic regulators [57]. HA enhances the generation of reactive oxidants and lipid peroxides, impairing cell viability and cell cycle progression in the presence of hydrogen peroxide in splenic mononuclear cells of a mouse breast cancer cell line [58]. The morphological alterations observed in RBCs in HA-exposed and recovered groups could be attributed to the osmotic power of HA, owing to its high-water binding capacity [9].

Culture of HA with ovarian cells of mice led to the production of gametes with compromised morphology [59]. Exhaustion of energy reserves (ATP) after fluid deficit or enlargement of the outer membrane layer, producing recognizable spicules, are involved in the creation of RBCs, followed by autophagy and auto lysosomal degeneration [60]. As the tear-drop cells travel through the splenic sinusoids or the bone marrow, they become compromised or deformed [23, 61]. The disturbance in the cholesterol/phospholipids ratio of the cell membrane underlies the presence of acanthocytes. Schistocytes are formed due to mechanical damage of RBCs by the fibrin strands present within the microvascular structure [44, 62]. The nuclear anomalies are due to chromosomal aberrations [52] and free radicals-mediated genotoxicity [63, 64]. The presence of erythrocytes with bilobed nucleus is associated with mitotic cell proliferation as a short-term compensatory method against hypoxia [28, 65].

The occurrence of elliptocytes, dacrocytes, and schistocytes indicates compromised splenic function in fish, likely attributed to factors including drug toxicity and exposure to industrial effluents [66].

The spleen, a significant peripheral lymphoid organ, plays a crucial role in antigen trapping [67]. Acting as one of the primary hematopoietic sites in teleost fish due to the absence of bone marrow cavities [68], the spleen is rich in lymphocytes and macrophages, pivotal components of the fish’s defense system [69, 70]. Despite its recognized importance in immune system regulation, the structure and microanatomy of the spleen have received comparatively less attention. Therefore, our study focused on studying the histological alterations that occurred in the spleen of *C. gariepinus* after HA exposure. Our finding demonstrated that exposure to HA cause numerous histological changes in the spleen of *C. gariepinus*, and the severity of these changes was dose-dependent in the exposed and recovered groups. *C. gariepinus* exposed to a low dose of HA showed mild histological alterations in spleen morphology with slight connective proliferation. Following recovery, these changes also persisted in *C. gariepinus* exposed to a medium dose of HA, showing moderate histological alterations in spleen morphology with increased connective proliferation around the wall of blood vessels and parenchyma. These alterations were also present to the same degree after recovery. However, *C. gariepinus* exposed to a high dose of HA exhibited severe pathological changes, including neutrophilic infiltration, lymphocytic exhaustion, and considerable connective tissue proliferation that extended to separate the splenic parenchyma into small compartments. Thickening of the walls of the blood vessels and splenic trabeculae were also clearly noticed in this stage. Recovery from high dose exposure did not prevent these deformities, but the changes were to a lesser extent than in the exposed group. The dose-related accumulation of collagen in the spleen could be due to increased blood lactate levels that activate transforming growth factor-beta, leading to fibroblast differentiation [71].

Our results were incompatible with those obtained by [72], who found that silver catfish (*Rhamdia quelen*) infected with *Citrobacter freundii* exhibited numerous changes in hematological parameters, including regenerative anemia, leukopenia, and an increase in melanomacrophage centers (MMCs) in the spleen and cephalic kidney of infected fish.

Under conditions of environmental stress, MMCs may increase in size or frequency and have been proposed as reliable indicators for assessing water quality, encompassing both oxygen depletion and iatrogenic chemical contamination [68].

The peroxidative damage under HA overload stimulates excessive formation of extracellular matrix proteins, ensuring an ideal environment for tissue fibrosis [73]. The observed histological alterations in the spleen could stem from its incapacity to sequester and expel HA as an exogenous xenobiotic substance, potentially impairing fish immune function. These results are in line with previous research by Sun [74], which documented splenic lesions, antioxidative responses, and inflammatory changes in catfish (*Pelteobagrus fulvidraco*) due to cadmium toxicity and also with Bardhan et al. [75] who studied the haematological and histologic changes of the spleen in *Oreochromis niloticus* after administration of florfenicol antibiotic.

## Conclusion

The findings of the current study are significant in drawing attention for the first time to the ecotoxicological effects of HA in aquatic creatures. The outcome measures could be suggested to decision-making authorities as diagnostic tools for the early detection of HA pollution during monitoring programs. Our findings confirmed the sensitivity of hematological indices, RBC morphological aspects, and splenic hemopoietic potential to HA in relation to its doses. Most of these abnormalities were still evident following the withdrawal period. Thus, HA should be taken into account in ecosystem assessment plans as a newly discovered hazardous element. Further investigations are strongly recommended to explore the other potential toxicological impacts of HA using dose/time response protocols.

## Data Availability

All relevant raw data will be freely available from the authors.
